# Does the late positive component reflect successful reading acquisition? A longitudinal ERP study

**DOI:** 10.1016/j.nicl.2017.10.014

**Published:** 2017-10-13

**Authors:** Christian Wachinger, Susanne Volkmer, Katharina Bublath, Jennifer Bruder, Jürgen Bartling, Gerd Schulte-Körne

**Affiliations:** Department of Child and Adolescent Psychiatry, Psychosomatics and Psychotherapy, Klinikum der Universität München, Ludwig Maximilian University Munich, Germany

**Keywords:** ERP, Event-related potentials, LPC, Late positive component, Dyslexia, Event-related potentials, EEG, Brain development, Longitudinal

## Abstract

Developmental dyslexia is a reading disorder that is associated with deficits in phonological processing, where the exact neural basis for those processing deficits remains unclear. In particular, disagreement exists whether degraded phonological representations or an impaired access to the phonological representations causes these deficits. To investigate this question and to trace changes in neurophysiology during the process of reading acquisition, we designed a longitudinal study with event related potentials (ERPs) in children between kindergarten and second grade. We used an explicit word processing task to elicit the late positive component (LPC), which has been shown to reflect phonological processing. A brain-wide analysis of the LPC with an electrode-wise application of mixed effects models showed significantly attenuated amplitudes in the left temporo-parietal region in dyslexic children. Since these differences were only present in the word and not in the picture (i.e. control) condition, the attenuated amplitudes might reflect impaired access to the phonological representations of words. This was further confirmed by the longitudinal development, which showed a rapid increase in amplitude at the beginning of reading instruction and a decrease with continuing automatization, possibly pointing to a progression from grapheme-phoneme parsing to whole word reading. Our longitudinal study provides the first evidence that it is possible to detect neurophysiological differences in the LPC between children with dyslexia and control children in both preliterate and very early stages of reading acquisition, providing new insights about the neurophysiological development and a potential marker of later reading problems.

## Highlights

• First longitudinal study of the late positive component (LPC)
• Left temporo-parietal LPC differs between control and dyslexic children.
• LPC increase at reading acquisition and decrease with automatization in typical readers.
• Dyslexic children do not show this neurophysiological course of development.
• Findings might indicate an impaired access to phonological representations.

## Graphical abstract

**Figure:**
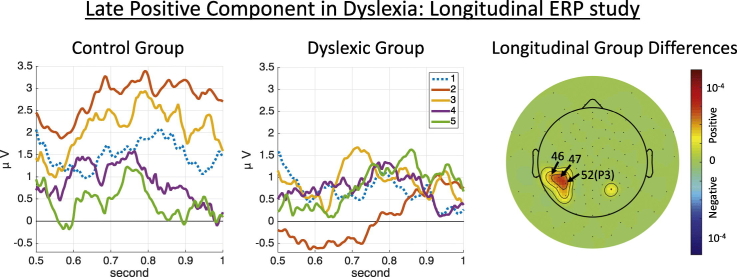

## 1 Introduction

Developmental dyslexia is one of the most common learning disorders, affecting 4–7.5% of all school age children (Fortes et al., 2016, Katusic et al., 2001, Landerl and Moll, 2010, Lewis et al., 1994). It is characterized by serious difficulties in learning to read and write in spite of sufficient cognitive ability and opportunity. Furthermore, the deficits are not the result of neurological, visual, or auditory impairment (APA, 2013). Numerous studies indicate that phonological processing deficits are a core deficit of impaired reading acquisition in dyslexia (Bradley and Bryant, 1983, Ramus et al., 2003, Torgesen et al., 1994, Vellutino et al., 2004). According to the phonological deficit theory, a specific deficit in dyslexia lies in the representation, storage and/or retrieval of speech sounds (Ramus et al., 2003). Brain imaging studies investigating auditory correlates of phonological processing in infants, kindergarten and school children learning to read further support the phonological deficit in dyslexia (Bonte and Blomert, 2004, Guttorm et al., 2005, Maurer et al., 2003, Molfese et al., 2002). One explanation for the phonological deficit is that the phonological representations of words are degraded in people with dyslexia, i.e., weak, coarse, unspecified or they contain too many allophonic details^1^ that are not relevant for distinguishing one word from another (Elbro, 1998, Goswami, 2015, Serniclaes and Sprenger-Charolles, 2003). An alternative explanation is that the phonological representations themselves are intact, but that the access to the representations in the phonological lexicon is impaired (Boets et al., 2013, Ramus, 2014, Ramus and Szenkovits, 2008). Consistent with the second assumption, Verhoeven et al. (2016) found that emerging literacy was primarily predicted by the accessibility of phonological representations in the mental lexicon and not by the specificity of those representations.

In neurophysiological studies with event-related potentials (ERP), phonological processing is primarily reflected in late components, in contrast to early components that mainly reflect basic visual or auditory processing. Particularly the access to the phonological representations seems to be reflected by the late positive component (LPC) with a latency of 500–900 ms (Hasko et al., 2013). Furthermore, the LPC has been related to word and pseudoword learning (Bermúdez-Margaretto et al., 2015, Perfetti et al., 2005). Attenuated LPC amplitudes have been reported for adults (Rüsseler et al., 2003), adolescents (Schulte-Körne et al., 2004), and children (Hasko et al., 2013) with dyslexia, as well as for subjects with low reading skills (Balass et al., 2010, Perfetti et al., 2005).

Common to all of these previous studies of the LPC is that they have reported on the outcome in lieu of the process of reading acquisition, i.e., they measured the LPC at one time point in participants who already have a diagnosis of dyslexia. Yet, dyslexia is a disorder that is characterized by difficulties in the developmental process of learning to read. Findings on older children and adults with dyslexia may therefore identify neural correlates that describe both deficient and compensatory brain processes at the same time. The potential confounding effects of compensatory brain processes can be reduced by investigating children during an early reading acquisition phase, mainly before and in the first grades of school, which is critical for the origination of dyslexia. Investigating dyslexic children in that developmental phase would allow us to observe whether different brain functions exist already before the onset of reading instruction or when during initial reading acquisition these differences emerge. In addition, preliterate children at risk for dyslexia have been found to show altered brain processes as early as at kindergarten age and even few days after birth (Guttorm et al., 2005, Maurer et al., 2003) as a possible consequence of their genetic predisposition (Scerri and Schulte-Körne, 2010). Current studies about the LPC in dyslexia have reported results for children in second grade (Hasko et al., 2013) or older.

Given the key role of phonological processing deficits in dyslexia, we designed a longitudinal LPC study that starts before the beginning of reading instruction and covers the key period of reading acquisition. This offers a unique opportunity to study the neural changes induced by learning to read at an age when phonological and visual abilities are already well developed. We recruited children with high risk for developing dyslexia and acquired ERP data at five time points: in kindergarten (T1), in the middle of first grade (T2), at the end of first grade (T3), in the middle of second grade (T4), and at the end of second grade (T5). This design results in four measurements after the onset of reading instruction and one at a pre-literate stage. This longitudinal design is well suited to identify developmental neurophysiological processes because changes within the same children are observed over time and it controls for intervening variables such as research methods or subject-dependent factors (Kraemer et al., 2000).

To elicit ERP responses that reflect the access to the phonological lexicon, we chose a task that requires decoding of visually presented words. Children were first presented a written word and had to decide whether it matched with an acoustically presented word. As control task, a picture was presented instead of the word. In the word condition, the access to phonological representations mainly takes place via grapheme-phoneme-conversion, in the picture condition via semantic information (Valente et al., 2016). This difference allows for potentially disentangling whether the representations are degraded (deviant ERPs in both conditions) or whether the access is impaired (deviant ERPs only in the word condition).

Previous longitudinal ERP studies with preschool children (Maurer et al., 2005, Maurer et al., 2007, Maurer et al., 2006, Lyytinen et al., 2004, Plakas et al., 2013) have focused on basic visual or auditory processing reflected by earlier ERP components (around 100–200 ms). Consequently, the employed ERP tasks differ from our task, which was chosen to elucidate the potential origin of the phonological deficit. Maurer et al., 2005, Maurer et al., 2007, Maurer et al., 2006 used an implicit word processing task to investigate print sensitivity. Children were presented with two successive visual stimuli and had to decide whether the first stimulus matched the second one. The results showed an impaired tuning for print in dyslexic children, measured by early ERP components. The Jyväskylä Longitudinal Study of Dyslexia (e.g. Lyytinen et al., 2004) and the Dutch Dyslexia Program (e.g., Plakas et al., 2013, van Leeuwen et al., 2008) investigated the significance of auditory processing as an early predictor of dyslexia. They showed that auditory processing in young children (measured by the mismatch negativity and other ERP components) differentiates those at risk of dyslexia from control children and is a possible predictor of later reading skills (for a review, see Volkmer and Schulte-Körne, in press).

Since this is the first longitudinal study analyzing the LPC, we follow a data-driven, mass-univariate approach with an electrode-wise analysis. This allows for localizing the effect without the need to formulate regions of interest a priori. We expected to find attenuated LPC amplitudes in children with dyslexia compared to the control children. Furthermore, we expected these group differences to be smaller at kindergarten age and to become more pronounced with reading acquisition. We differentiate across- and within-subject variations with mixed effects models and correct for multiple comparisons. The results indicate a deviant access to the phonological representations in dyslexia during the process of reading acquisition displayed as an attenuation of the LPC in the left temporo-parietal region. Interestingly, these differences are already present at kindergarten and vanish at the second grade with the continuing automatization of reading.

## 2 Materials and methods

### 2.1 Participants

The sample was recruited by contacting parents of preschool children at parent-teacher conferences in elementary schools (at time of enrolment) and kindergartens in and around Munich, as well as from special schools for reading impaired children and the German Dyslexia and Dyscalculia Association (Bundesverband Legasthenie und Dyskalkulie e. V.). A particular challenge for implementing a longitudinal study on early predictors of dyslexia that starts before reading instruction is the recruitment, since it is not clear at the beginning of the study which participants will develop dyslexia. We included a large percentage (58%) of children with familial risk of dyslexia in the study with the expectation of having a higher number of dyslexia cases than the population average. Thus, two groups of children were recruited: Children with a familial risk of dyslexia (at least one parent or sibling with dyslexia or reading/writing difficulties) and children without such a risk (control group). In total, 86 monolingual kindergarten children were recruited. All parents signed an information consent form and completed a questionnaire regarding reading and spelling difficulties in their families.

Exclusion criteria were the following: Children who were already able to read simple monosyllabic words (nouns) in kindergarten (n = 6), exceeded the cut off score for ADHD (score > 7) (n = 4) or were unable to cooperate during any of the ERP recordings (e.g. did not fixate on the screen) (n = 3). At the end of second grade the sample consisted of 64 children (9 children dropped out). All subjects had normal hearing, and normal or corrected-to-normal vision.

### 2.2 Design and procedure

All participants were tested at five time points: in kindergarten (T1, mean age 6.2), in the middle of first grade (T2, mean age 6.9), at the end of first grade (T3, mean age 7.2), in the middle of second grade (T4, mean age 7.8), and at the end of second grade (T5, mean age 8.2). In Germany, children do not receive formal literacy instruction until they enter primary school at the age of six years. Some children may nevertheless have picked up elements of reading or are even able to decode words before entering primary school (Rückert et al., 2010, Rückert and Schulte-Körne, 2010, Sénéchal and LeFevre, 2002). Therefore, a non-standardized test of letter knowledge and a non-standardized test of reading simple words were conducted with the kindergarten children. The letter knowledge test measured the number of all German letters known: Letters were presented individually one per card and the children had to name the letter. During the reading test the children had to read four monosyllabic nouns. If they were able to read one of the four words, they were not included in the study, as the first measurement point was intended to assess pre-(non-)reading children. Furthermore, a non-verbal intelligence test (Bulheller and Häcker, 2002), a questionnaire for Attention Deficit Hyperactivity Disorder (ADHD) (Achenbach, 1991), and a handedness questionnaire (Schulte-Körne et al., 1998) were conducted in kindergarten.

Reading instruction in Germany is mainly phonics based. It starts when children enter primary school (at the age of six years), with the instruction of single letters and includes much practice at syllable level. It takes nearly till the end of first grade until children have learned all graphemes and corresponding phonemes. However, reading accuracy is already high at this time point and children merely improve in reading speed from then on (Landerl and Wimmer, 2008). Reading speed therefore differentiates well between good and poor readers with large deficits for children with dyslexia (Ferrer et al., 2015, Landerl and Wimmer, 2008). For this reason, we tested the children at the end of second grade (T5) with a standardized reading fluency test to diagnose dyslexia: The one-minute reading fluency test from the SLRT-II mainly assesses reading speed, but also takes into account the error rate (Moll and Landerl, 2010). Children were presented first a word and then a pseudoword list. Within 1 min, they read aloud as many words and pseudowords as they can. The number of correctly read words is the raw score of the test. At the end of second grade, when the test was conducted, the diagnosis of dyslexia is already highly reliable.

In accordance with the literature (Gallagher and Frith, 2000, Lyytinen et al., 2006, Schulte-Körne et al., 1996), we expected 50–70% of the at-risk children to develop reading difficulties. However, only 10% of the at-risk children showed later word and pseudoword reading fluency that fell in the range of our dyslexia diagnosis. Therefore, new groups were defined based on reading fluency at T5 and the familial risk was added as variable to our analyses. The dyslexia group consisted of 16 children, the control group of 15 children. Both groups had a mean non-verbal IQ within the normal range and did not differ in age. Furthermore, gender and handedness did not differ significantly between the groups, as shown in Table 1. The average letter knowledge in the control group is 15.8 ± 7.3 and in the dyslexia group 12.8 ± 6.4, where the difference is not statistically significant.

**Table 1:** Descriptive statistics of participants.

	Whole sample
N	64
Handedness (r/l)	57/7
Gender (m/f)	36/28
Age T1	6.1 (0.4)
IQ T1	108.3 (11.6)


	Dyslexia	Control
n	16	15
Handedness (r/l)	15/1	12/3
Gender (m/f)	10/6	10/5
T1		
Age	6.2 (0.4)	6.2 (0.3)
IQ	108.8 (10.9)	107.4 (11.8)
Letter knowledge (T1)	12.8 (6.4)	15.8 (7.3)
T2		
Age	6.9 (0.4)	6.8 (0.3)
Word reading fluency⁎⁎	8.2 (6.0)	15.1 (7.3)
Pseudoword reading fluency⁎⁎	10.8 (6.0)	18.1 (5.8)
T3		
Age	7.2 (0.4)	7.2 (0.4)
Word reading fluency⁎⁎⁎	17.2 (8.7)	39.2 (11.6)
Pseudoword reading fluency⁎⁎⁎	15.2 (5.5)	28.2 (4.6)
T4		
Age	7.8 (0.3)	7.8 (0.3)
Word reading fluency⁎⁎⁎	25.8 (9.1)	56.4 (12.3)
Pseudoword reading fluency⁎⁎⁎	20.8 (4.8)	36.3 (8.7)
T5		
Age	8.2 (0.4)	8.2 (0.4)
Word reading fluency ⁎⁎⁎	28.1 (6.5)	67.1 (14.7)
Pseudoword reading fluency ⁎⁎⁎	18.7 (4.8)	38.3 (5.6)

Note. Standard deviations appear in parentheses.

⁎⁎ *p* < 0.01.

⁎⁎⁎ *p* < 0.001.

### 2.3 Procedure

**Fig. 1:**
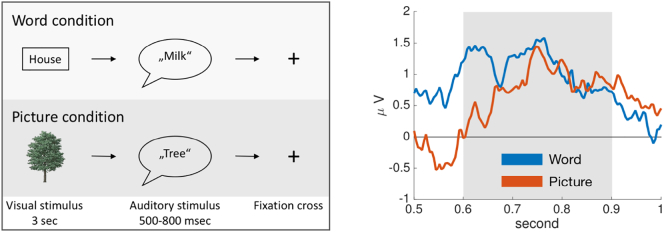
*Left*: The ERP paradigm consists of a word and a picture processing task. Each task begins with a visual stimulus followed by an auditory stimulus. At the fixation cross the children decide whether it is a congruent or an incongruent pair. *Right*: Illustration of average EEG recordings in the control group for the word and picture condition. As a measure of the late positive component, we average the activity in the window from 600 to 900 msec.

The ERP paradigm consists of a word processing task in the experimental condition and a picture processing task in the control condition, see Fig. 1 (left). Picture processing is thought to activate similar processes as word processing such as conceptualization, selection of a lexical item and phonological encoding (Levelt et al., 1998). The main difference between the picture and the word condition is that word reading requires the decoding of graphical and orthographical material, respectively. Thus, both conditions need to access phonological representations, although the way of access is different in the word condition as it comprises orthography.

In the word condition, the children were presented a word (3 s) on the screen followed by an auditory presentation (500–800 ms) of a word that either sounded like the presented visual stimulus (congruent) or did not sound like it (incongruent). Subjects had to decide if they thought the pair was congruent or incongruent and respond by button press. The picture condition was analogous to the word condition: children had to decide if the picture they saw was congruent with the subsequently spoken word (Bublath, 2010). Retrieving the phonological information of the visual input is necessary for the subsequent matching task. Kindergarten children were encouraged to imagine the sound of known letters in the presented word because they could not read yet.

Children were given 5 s for their decision, during which a fixation cross was displayed. The next stimulus appeared immediately upon button press. In the case of no decision within the five allotted seconds, the next stimulus was presented. For each condition, 40 congruent and 18 incongruent items were presented, totaling 58 items. For both conditions, 116 visual stimuli and 116 auditory stimuli were presented per participant. The incongruent items were utilized to guarantee an interesting and varied task. The experiment was divided into eight blocks with alternating picture or word conditions to rule out block-order effects. All words and picture-names (4–5 letters long) were taken from the basic primary school vocabulary and from the basic vocabulary by Pregel and Rickheit (1987). This ensured a selection of word and picture material with a high probability of frequent verbal usage and visual contact by children in our age range. The words and pictures were presented on a 17-inch computer screen placed about 1 m in front of the children. Words were shown in capital letters of the font Arial with font size 75. The vertical angle was 2.3 degrees and the horizontal angle was 8.9 degrees in the word condition. In the picture condition the vertical and horizontal angles were both 11.5 degrees. Spoken words (female voice) were recorded with the computer program CoolEdit and were presented with Sennheiser PX200 headphones (~ 70 dBA). The duration of the whole experiment, including instructions and testing, was 30–40 min.

### 2.4 Data recording and analysis

EEG was recorded during the visual and the auditory stimulus presentation with an Electrical Geodesic Inc. 128-channel-system. The impedance was kept below 50 kΩ. EEG-data were recorded continuously with Cz as the reference electrode and sampled at 250 Hz. Filtering (highpass 0.3 Hz, lowpass 30 Hz, phase-shift free Butterworth filters with 12 dB/octave, filtered continuous on raw data to avoid discontinuities and transient phenomena) and electrooculographic artifact removal with Independent Component Analysis (Zhou et al., 2005) was performed with Brainvision Analyzer (Brain Products GmbH). The EEG data was re-referenced to the average reference and segmented into epochs spanning 1100 ms intervals, where 100 ms are pre-stimulus and 1000 ms are post-stimulus. We included all 58 trials in the analysis, as at the time of the presentation of the visual stimuli congruent and incongruent do not differ. The further data analysis was performed in Matlab (MathWorks Inc.), where electrodes per trials were excluded according to the following three criteria: (i) gradient criterion: > 50 μV difference between two successive data points or > 150 μV in a 200 ms window, (ii) absolute amplitude criterion: more than ± 150 μV, (iii) low activity criterion: < 0.5 μV difference in a 100 ms window. For the word condition 4.85% of data was rejected and for the picture condition 4.72%. Finally, the data was baseline corrected and averaged per condition, time point, and subject.

Fig. 1 (right) illustrates the late positive component for the word and picture condition in control subjects. As the LPC does not show a clear peak, we computed the average amplitude in the window from 600 to 900 ms, according to the inspection of the global field power and the definition of the LPC time window in previous work (Hasko et al., 2013, Van Strien et al., 2011). The ERP waveforms in the left temporo-parietal region for both conditions are shown in Supplementary Fig. 1 and for both groups in Supplementary Fig. 2. The topographic map of the late component for both conditions is plotted in Supplementary Fig. 3.

### 2.5 Statistical analysis

We employed linear mixed effects models (Verbeke and Molenberghs, 2009) to study the longitudinal development of the LPC that have, for instance, previously been applied to analyze longitudinal brain morphology (Wachinger et al., 2016). A separate model was fitted for each electrode, where we restricted the analysis to electrodes with a positive late component, resulting in 58 out of 129 electrodes. Negative components were mainly present in the right frontal and right inferior temporal regions, as seen in the topographic map, which are of no further interest to this study. Due to multiple comparisons, a correction for the false discovery rate (FDR) at *q* = 0.05 was used (Benjamini and Yekutieli, 2001). As ERP input to the model, we computed the difference between the word and picture condition of the LPC, where the LPC is defined as the average amplitude across trials in the 600–900 ms window. In the first model, we analyzed the longitudinal changes in the LPC during reading acquisition. Given the time-from-baseline *X*_*ij*_ for individual *i* at follow-up scan *j* and the LPC difference *Y*_*ij*_ as dependent variable, the resulting model is

(1) $$Y_{\mathrm{ij}}\;=\beta_{0}+\beta_{1}X_{\mathrm{ij}}+\beta_{2}A_{i}+\beta_{3}F_{i}+b_{i},$$

where *β*_0_, …, *β*_3_ are fixed effects regression coefficients and *b*_*i*_ is a random effect regression coefficient. The random effect enables modeling individual-specific intercept. Next to the main effect, we include age *A*_*i*_ and familial risk *F*_*i*_ in the model and perform the analysis on children in the control group (n = 15).

In the second model, we analyzed the differences in the longitudinal development between the control (n = 15) and dyslexic (n = 16) group. We included the group *D*_*i*_ (control or dyslexia) together with interaction with the time-from-baseline to the model, yielding

(2) $$\begin{array}{cc} Y_{\mathrm{ij}} & =\beta_{0}+\beta_{1}D_{i}+\beta_{2}X_{\mathrm{ij}}+\beta_{3}D_{i}X_{\mathrm{ij}}+\beta_{4}A_{i}+\beta_{5}F_{i}+b_{i}, \end{array}$$

where *β*_0_, …, *β*_5_ are fixed effects regression coefficients. The control group serves as reference category (baseline level) in the model. We performed an additional analysis with a variation of the second model, where we replaced the dichotomous group variable *D*_*i*_ with the number of correctly read words from the SLRT (continuous) in the model. This additional analysis was not restricted to the control and dyslexia groups, but involved all children (n = 64).

Overall, we had measurements for five time points from kindergarten to second grade, which reflected different stages of reading acquisition. We split the analysis in two time segments to have a more homogeneous development in each of the segments. The first segment, with time points 1 and 2, reflects the process of initial reading acquisition. The second segment, with time points 2 to 5, reflects increasing automatization in reading.

## 3 Results

### 3.1 Behavioral data

Next to the development in reading fluency, we studied group differences in the proportion of correctly answered trials. Table 2 reports the average number of correctly matched visual and auditory stimuli over 58 trials for all time points and both groups. In the picture condition, there were no group differences in any of the measurement points. In the word condition, the control group matched the written word significantly more often to the following auditory stimulus at T2 (*p* = 0.037) and T3 (*p* < 0.001) than the dyslexia group. At T1, T4 and T5, no group differences existed. The missing group differences at T4 and T5 were probably due to ceiling effects. The word material (identical across time points) became very easy in second grade and the response accuracy does therefore not differentiate between good and weak readers anymore. We measured no significant differences in reaction time between both groups. In the ERP analysis, trials with correct and incorrect answers were included.

**Table 2:** Mean and standard deviation for correct answers in the ERP task for all time points and both groups. For each condition, 58 trials were performed.

	T1		T2		T3		T4		T5	
Condition	Word	Pict.	Word⁎	Pict.	Word⁎⁎⁎	Pict.	Word	Pict.	Word	Pict.
Dyslexia	33.3 (9.5)	35.9 (6.6)	50.3 (6.2)	57.1 (0.7)	54.3 (3.1)	56.1 (2.2)	56.9 (1.6)	56.7 (1.0)	56.1 (1.4)	56.9 (1.3)
Control	37.5 (9.9)	36.8 (5.4)	55.3 (2.9)	56.7 (1.4)	56.7 (1.0)	56.5 (1.8)	56.3 (1.8)	56.8 (1.3)	56.2 (1.8)	56.3 (1.9)

Note. Standard deviations appear in parentheses.

⁎ *p* < 0.05.

⁎⁎⁎ *p* < 0.001.

### 3.2 Event related potentials

**Fig. 2:**
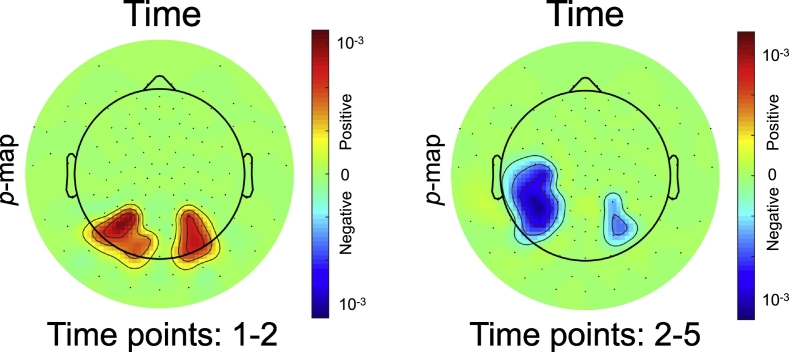
P-maps of the *time* variable in the LME model for analyzing the LPC. Non-significant regions are shown in green. Significant regions with positive regression coefficients are illustrated in red and analogously regions with negative regression coefficients in blue. Colorbar shows FDR corrected p-values. (For interpretation of the references to color in this figure legend, the reader is referred to the web version of this article.)

We first analyzed longitudinal changes in the LPC during normal reading development in controls with the model in Eq. (1). Fig. 2 illustrates the results as a topographic map of *p*-values for time points 1–2 and time points 2–5, where a separate LME model was computed for each electrode. Statistically significant regions after FDR correction are shown in red or blue, where more saturated colors indicate higher significance. To also encode the direction of the regression coefficient in the *p*-map, we indicate positive regression coefficients in red (i.e., an increase in amplitude with time) and analogously negative regression coefficients in blue. With the beginning of reading instruction, captured by time points 1 to 2, the amplitude increased. With continuing reading instruction, the amplitude decreased. The effect was present on both hemispheres in the occipito-parietal region and slightly more pronounced in the left hemisphere.

**Fig. 3:**
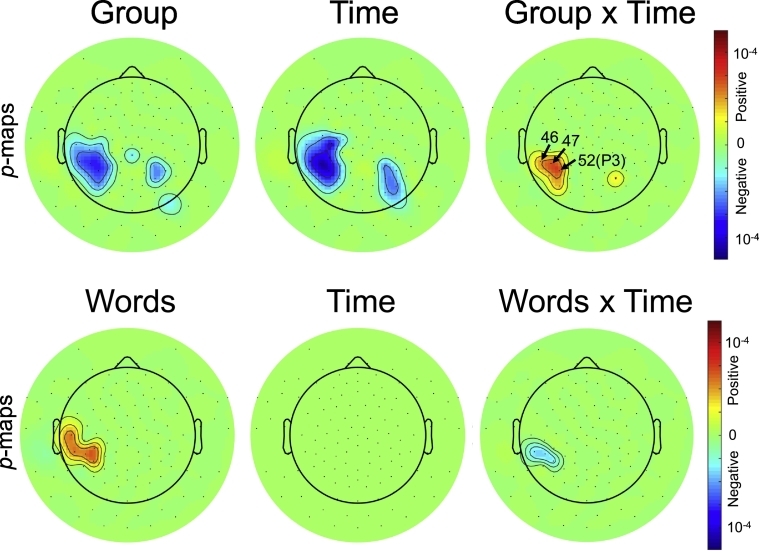
P-maps of the main effects in the LME model for analyzing the LPC for time points 2 to 5. Results are shown for the LME model with the *group* variable (top) and the number of correctly read *words* (bottom). Non-significant regions are shown in green. Significant regions with positive regression coefficients are illustrated in red and analogously regions with negative regression coefficients in blue. Electrodes 46, 47, and 52 (P3) are labeled in the image. Colorbar shows FDR corrected *p*-values. (For interpretation of the references to color in this figure legend, the reader is referred to the web version of this article.)

In the next analysis, we used the LME model in Eq. (2) to identify differences in LPC between control and dyslexic group during reading acquisition. Fig. 3 shows *p*-maps for the main effects *group* and *time* together with their interaction for time points 2 to 5, where the control group was encoded as baseline in the model. The dyslexic group had a lower amplitude than the control group, shown by the negative regression coefficients for the *group* variable. The negative regression coefficients for the *time* variable indicate a decrease of amplitude with reading instruction. The positive coefficients for the interaction *group* × *time* indicate an opposite change with time for the dyslexic group. To summarize, the control group started with a higher amplitude, which decreased over time points; in contrast, the dyslexic group started with a lower amplitude, which increased over time points. The effect was mainly localized in the left temporo-parietal region. Supplementary Fig. 4 shows *p*-maps for the word and picture conditions separately, instead of the difference between both conditions. The LPC of the picture (control) condition did not show any significant effects, whereas the results of the word condition were consistent with the results of the difference between both conditions.

In Fig. 3, we also show the results for the variation of the second model, where the group variable was replaced with the number of correctly read words (SLRT) and all children were included. The results were consistent with the group analysis. The negative coefficients for the interaction of *words* × *time* indicated a decrease in amplitude for good readers, which started off with a higher amplitude as indicated by the positive effect of *words*. The effect of *time* is not significant after FDR correction although a positive effect was present at a less strict significance level of *p < 0.01.*

Consistently across both analyses, the strongest interactions were present at electrodes 46, 47, and 52, labeled in Fig. 3. Table 3 lists the exact characteristics of the LME model for these electrodes. The table reports uncorrected *p*-values, whereas corrected p-values are shown in the figures.

**Table 3:** Standardized regression coefficients for the LME model and un-corrected p-values in parentheses for three electrodes with largest effects. Results are shown for *group* × *time* and *words* × *time* models, which are an excerpt from the same results visualized in Fig. 3. *P*-values are rounded to four decimals places. Numbers are shown in bold face if they reach significance after FDR correction. Age and familial risk are not included in the table as they are not significant.

Electrode	Group	Time	Group × Time
47	**− 1.796 (0.0000)**	**− 0.465 (0.0000)**	**0.533 (0.0000)**
46	**− 1.660 (0.0000)**	**− 0.395 (0.0000)**	**0.484 (0.0001)**
52	**− 1.723 (0.0000)**	**− 0.443 (0.0000)**	**0.528 (0.0000)**


Electrode	Word	Time	Word × Time
47	**0.031 (0.0002)**	0.335 (0.0150)	**− 0.010 (0.0004)**
46	**0.029 (0.0006)**	0.351 (0.0120)	**− 0.009 (0.0009)**
52	**0.027 (0.0009)**	0.237 (0.0827)	**− 0.008 (0.0022)**

In a post-hoc analysis, we evaluated whether significant group differences in the left temporo-parietal region had already been present at kindergarten (first time point). We noted significant group differences for electrodes 47 (− 1.958, *p* < 0.05), 51 (− 2.617, *p* < 0.05), and 52 (− 2.494, *p* < 0.01). A similar analysis with reading fluency instead of diagnostic group in the model also showed significant associations between LPC and correctly read words for electrodes 46 (0.015, *p* < 0.05), 47 (0.021, *p* < 0.005), 51 (0.016, *p* < 0.05), and 52 (0.021, *p* < 0.005).

**Fig. 4:**
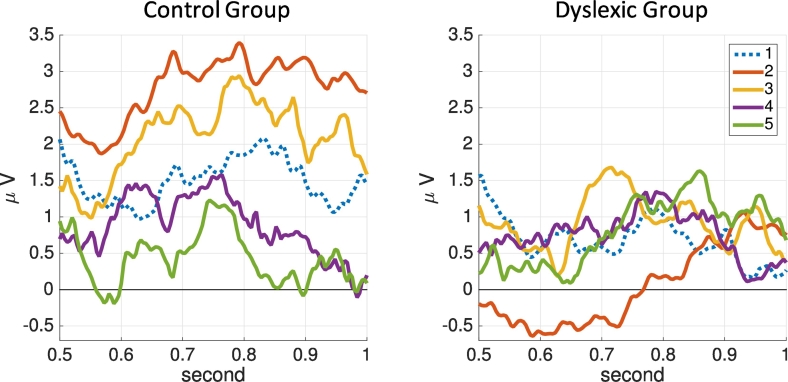
ERP waveforms of the LPC for electrode 47 for all five time points. The lines show averages over the control group (left) and dyslexic group (right) for the word condition. Dotted blue line: kindergarten before the beginning of reading instruction (T1), orange: middle of 1st grade (T2), yellow: end of 1st grade (T3), purple: middle of 2nd grade (T4), green: end of 2nd grade (T5). (For interpretation of the references to color in this figure legend, the reader is referred to the web version of this article.)

To illustrate the impact of reading instruction on the LPC, we plot average ERP waveforms of the word condition for all five time points for both groups in Fig. 4. The illustration is for electrode 47, which showed the strongest effects in the previous analyses. For the control children, the ERP amplitude increased from the first to the second time point. Interestingly, the amplitude then decreased continuously from the second to the fifth time point. In contrast, the ERP signal for children with dyslexia did not show such a development but rather stayed at a similar level except for a decrease at the second time point.

**Fig. 5:**
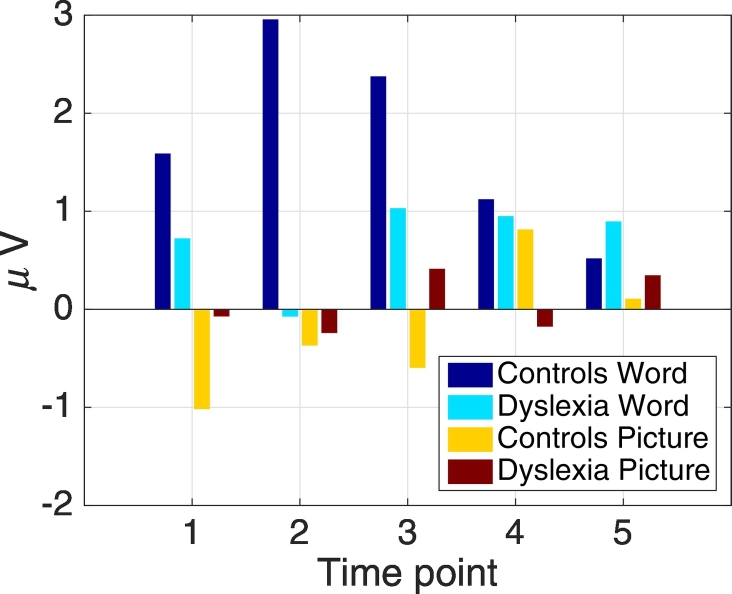
Averages of the LPC in the 600–900 ms window as bar plots for both groups and conditions. Illustrated for all five time points for electrode 47.

Finally, Fig. 5 shows the average LPC for both, the picture and the word condition, across groups. For the controls in the word condition, the amplitude strongly increased from the first to the second time point and then decreased for the remaining time points. The strong amplitude difference between the control and dyslexic group at the second time point was consistent with the negative *group* regression coefficient of the LME model in Fig. 3. The amplitude decrease in the control group from time points 2 to 5 was consistent with the negative regression coefficient of the *time* variable. In contrast, the dyslexic group showed an increase in amplitude for time points 2 to 5, consistent with the positive *group* × *time* interaction in the model. The picture condition showed small amplitudes that are not significant.

## 4 Discussion

To our knowledge, this is the first longitudinal LPC study. The LPC is important for the process of reading, as it probably reflects the access to the phonological representations of words (Hasko et al., 2013). Our longitudinal design captures the critical phase of reading acquisition with four time points in first and second grade. In addition, ERP data at kindergarten allows for assessing neurophysiological correlates before reading instruction. Children performed an explicit word processing task during the EEG acquisition, which is novel for a longitudinal ERP study. The primary aims of this study were to investigate neurophysiological changes during reading acquisition and alterations in the reading process for children with dyslexia.

Our results on non-dyslexic children in Fig. 2 showed an increase in amplitude of the LPC from the first to the second time point, i.e., from kindergarten to first grade. The effect was localized in the parietal region of the brain with a stronger effect on the left than on the right hemisphere. Such an increase in LPC amplitude has repeatedly been found to be associated with word recognition accuracy: The more words children identified correctly, the higher the memory-effect, i.e., the increase in LPC amplitude for recently viewed and recognized words (Van Strien et al., 2011). Word learning has shown to be similarly correlated to an increase in LPC amplitude. Furthermore, skilled readers, who were more accurate in meaning judgements on recently trained words, showed a stronger memory effect than less skilled readers (Perfetti et al., 2005). Bermúdez-Margaretto et al. (2015) also found that repeated exposure to pseudowords increased the LPC amplitude of young adults (correlating with an increase in pseudoword reading speed and accuracy), until they reached the amplitude of words. The LPC may therefore be related to the formation and strengthening of memory traces for word-like material. The authors assumed that the memory traces involve sub-lexical units at the beginning of the task and representations of whole words at the end, and further that these traces involve phonological rather than visual representation. Although word (or pseudoword) learning experiments cannot be equated with the process of initial reading acquisition, the increase of the LPC amplitude seems to be associated with both.

From the second to the fifth time point, we noted a decrease in amplitude, which is consistent with a priori expectations that additional neural activity is required for learning to read and that the activity decreases in the following with increasing automatization of the reading process. A similar decrease with ongoing repetition has been observed for familiarization with new words in young children: After an initial phase of increase in ERP amplitude, it decreased with further repetition, but only in children with a large productive vocabulary (Borgström et al., 2015, von Koss Torkildsen et al., 2009). This longitudinal development with an increase in activity at the beginning of reading instruction and a decrease with continuing automatization can possibly point to a progression from grapheme-phoneme parsing to whole word reading.

In contrast, the development of the LPC in the dyslexic group was significantly different. The dyslexic group exhibited a lower LPC amplitude in first grade, as shown by the negative coefficients of the *group* variable in Fig. 3 and Table 3. Further, the amplitude of the dyslexic group increased with time, while it decreased for the control group. The analysis with reading fluency in Fig. 3 confirmed the results from the group analysis. The previously described pattern during normal reading acquisition of increasing amplitude followed by a decrease with automatization was therefore not present in the dyslexic group. The group differences were primarily in the left temporo-parietal region. Altered activity in the left temporo-parietal regions on tasks of word reading have previously been reported in dyslexia (Temple et al., 2001, Simos et al., 2000b). It is associated with the mapping of graphemes of a visual word onto the phonological representation as it lies on the dorsal pathway that includes the angular and supramarginal gyri, and also the left posterior end of the superior temporal gyrus (Simos et al., 2000a). Interestingly, we did not find group differences in the picture control condition, although pictures also need to be mapped to phonological representations to complete the task. Yet, the access to the representations for both conditions is different: In the word condition the access mainly takes place via grapheme-phoneme-conversion, in the picture condition via semantic information (Valente et al., 2016). Our results therefore support the hypothesis of an impaired access to phonological representations in dyslexia (Boets et al., 2013, Ramus, 2014, Ramus and Szenkovits, 2008). The impaired access has been associated to differences in functional connectivity patterns between Broca's area and the left superior temporal gyrus together with structural connectivity differences in the left arcuate fasciculus. Our ERP results of the LPC provide an alternative view on the impaired access in dyslexia and, importantly, assess the LPC development during the process of reading instruction. The increase in activity in the control group together with the subsequent decrease with continued reading instruction points to the creation of connections that become more and more automatized. In contrast, these connections may not be created by dyslexic children, causing a continued disruption in the access to phonological representations.

In our analysis, we did not follow the traditional approach of defining a region of interest in which the ERP activity is averaged. Instead, we computed linear mixed effect models for each electrode. This has the unique advantage of clearly showing the spatial extent of the effect without the need for a manual region definition, particularly interesting for high-resolution EEG systems with 128 channels. Compared to previous cross-sectional LPC studies where parietal (Schulte-Körne et al., 2004) or centro-parietal (Hasko et al., 2013) regions of interest have been identified, we observe the most significant group differences in the temporo-parietal region. A challenge for this data-driven, mass-univariate approach is that spurious effects could be detected due to multiple comparison. We applied a conservative correction for the false discovery rate (Benjamini and Yekutieli, 2001) and obtained highly significant effects, although we only had 15 and 16 children per group, respectively, which clearly speaks for the large difference in LPC between the groups.

We split the analysis into time points 1–2 and 2–5. From Fig. 5 it is evident that a linear model across all time points would not reflect the data. An alternative would be the increase of the model complexity by adding a squared time-from-baseline term *X*^2^. However, this would make the interpretation of the model more challenging. In addition, the data does not seem to follow a quadratic distribution either. We therefore split the data in acquisitions that reflect the change from kindergarten to the beginning of reading instruction, followed by continuing reading instruction.

Our results provide a better understanding of neurophysiological development during the process of reading acquisition reflected by the LPC and its deviation in dyslexia. The longitudinal development of the LPC and the localization of differences in the temporo-parietal region has not been described before. The described pattern might help in designing prevention programs for dyslexic children, as our results suggest that dyslexia is probably rather associated with an impaired access to the phonological lexicon instead of degraded representations in this lexicon. Moreover, by investigating children at very early reading stages it was possible to analyze LPC deficits that are not confounded with compensatory brain activities as suggested from studies with older subjects (Georgiewa et al., 1999, Shaywitz et al., 2002). Therefore, our longitudinal study provided the first evidence that it is possible to detect neurophysiological differences in the LPC between children with dyslexia and control children in both preliterate and very early stages of reading acquisition. The post-hoc analysis showed that differences in the access to the phonological lexicon between the control and dyslexia group already exist in kindergarten, where previous studies support the existence of a phonological lexicon before literacy acquisition (Ainsworth et al., 2016, Yeh et al., 2015). Thus, LPC attenuation in young children might be understood as an early candidate predictor of later reading problems, before the start of formal reading instruction.

## Appendix A Supplementary data

Supplementary figures Image 2

## Footnotes

1 Allophones are variants of a phoneme which do not change the meaning of a word.
