# Supplementary material for: Does the late positive component reflect successful reading acquisition? A longitudinal ERP study
Source: Neuroimage Clin. 2017 Oct 13;17:232–40. doi: 10.1016/j.nicl.2017.10.014 (PMC5683196; doi:10.1016/j.nicl.2017.10.014)
Supplement: Supplementary file 1 — Supplementary figures [file mmc1.docx]

Supplementary Figure 1: ERP waveforms for all five time points averaged across all participants and electrodes in the left temporo-parietal region (46, 47, 50, 51, 52). Plots are shown for the word (left) and picture (right) condition.

Supplementary Figure 2: ERP waveforms for all five time points averaged across electrodes in the left temporo-parietal region (46, 47, 50, 51, 52). The lines show averages over the control group (left) and dyslexic group (right) for the word condition.

Supplementary Figure 3: Topographic plot of the late component (average of 600-900 ms) for the word and picture condition. In this study, we are interested in the late positive component, so that we limit the analysis to positive regions of the word condition.

Supplementary Figure 4: P-maps of the main effects in the LME model for analyzing the LPC for time points 2 to 5. Results are shown for the LME model with the *group* variable. In contrast to Figure 3, where the results of the difference between the word and picture condition are shown, this figure shows the results for word (top) and picture (bottom) separately. Non-significant regions are shown in green. Significant regions with positive regression coefficients are illustrated in red and analogously regions with negative regression coefficients in blue. Colorbar shows FDR corrected p-values.
